# Lower Toxicity of the Essential Oils With Repellent Potential Compared to Diethyltoluamide and Cypermethrin on *Porcellio laevis*

**DOI:** 10.1155/jt/6638848

**Published:** 2025-12-30

**Authors:** Heber Silva-Díaz, Angie Vilma Serrato-Monja, Emma Vanesa Arriaga-Deza, Lizzie Karen Becerra-Gutiérrez

**Affiliations:** ^1^ Faculty of Human Medicine, University of San Martín de Porres, Chiclayo, Peru, usmp.edu.pe; ^2^ Research Department, Lambayeque Regional Hospital, Chiclayo, Peru; ^3^ Faculty of Biological Sciences, Pedro Ruiz Gallo National University, Lambayeque, Peru

**Keywords:** DEET, insect repellents, isopoda (MeSH-NLM), oils, toxicity, volatile

## Abstract

**Objective:**

To evaluate the acute toxicity of essential oils with repellent potential, diethyltoluamide (DEET), and cypermethrin on *Porcellio laevis.*

**Methods:**

Randomized preclinical trial with a factorial and controlled arrangement on three essential oils (*Eucalyptus globulus*, *Mentha piperita*, and *Cymbopogon citratus*) at 0.1%, 1%, and 10%, respectively, DEET at 10% and cypermethrin at 0.1%. Each experimental group consisted of 10 specimens, 2–3‐mm‐long, of *P. laevis*. Toxicity was measured by specimen mortality at 3, 24, and 48 h postexposure. Nonparametric inferential statistics were used to compare mortality between the groups. The InfoStat/E software, Version 2020, was used for analysis.

**Results:**

Essential oils at concentrations of 0.1% and 1% showed similar toxicity to each other (mortality of 10%–20%) but significantly lower compared to cypermethrin and DEET (mortality of 100%). However, essential oils at 10% reached median mortality rates above 70%. Likewise, similar effects were observed at concentrations of 0.1% and 1.0% and at 24 and 48 h. The LC 50 at 24 h was 7.8% (CI 95%: 5.2–9.8), 6.1% (CI 95%: 4.9–7.4), and 9.8% (CI 95%: 8.9–10.6) for *E. globulus*, *M. piperita*, and *C. citratus,* respectively.

**Conclusions:**

The evaluated essential oils showed lower acute toxicity compared to DEET and cypermethrin, depending on concentration and time.

## 1. Introduction

Arboviral diseases, especially dengue, have increased alarmingly in recent years in tropical and subtropical regions. According to the World Health Organization (WHO) report, more than 4 billion people worldwide are at risk, and it is estimated that by 2050, the number will reach 5 billion. In fact, in 2023, cases of dengue doubled to more than 12.3 million by the end of August, classifying it as a Level 3 emergency [1].

The use of repellents is currently an effective and economical method for the prevention of arboviral diseases [2], with the synthetic compounds diethyltoluamide (DEET), picaridin, p‐methanediol (PMD), diethyl phenylacetamide (DEPA), and ethyl butylacetylaminopropionate (IR3535) [3] standing out. Of these, DEET is the most widely used commercially, with more than 200 million applications each year; however, significant adverse effects have been reported at the nervous system and skin level due to its toxicity and excessive use in humans [4, 5].

For this reason, it is convenient to evaluate other repellent options of natural origin, biodegradable, and selectively effective, but with less toxicity or adverse effects compared to DEET [6]. In this regard, several plant species have been used ancestrally for repellent purposes, mainly from the families Asteraceae, Cupressaceae, Lamiaceae, Lauraceae, Myrtaceae, Meliaceae, Poaceae, Piperaceae, Umbelliferae, Rutaceae, and Zingiberaceae [7], which contain different aromatic and aliphatic compounds that make them repellent, and in some cases, insecticidal and acaricidal activity [8]. In Cymbopogon citratus, the main compounds are coumarins, tannins, anthraquinones, and saponins [9]; in Eucalyptus globulus, it is 1,8‐cineole (eucalyptol) [10,11]; and in Mentha piperita, they are menthol, menthone, neomenthol and isomenthol [12]. These oils are effective against *Culex* and *Anophele*s mosquitoes [3, 7], and *Aedes aegypti* [2].

Indeed, previous studies have reported that the essential oils from *E. globulus*, *C. citratus*, and *M. piperita* to be effective repellents and have adequate times of protection against the *A. aegypti* mosquito, the transmitter of dengue, Zika, chikungunya, and other arboviral diseases [2, 13]. However, safety at the concentrations at which efficacy has been demonstrated is still unclear, partly due to the challenges of conducting safety trials on botanical active ingredients because they need invertebrate animal models [7, 14]. Among these models, the isopods of the genus *Porcellio* stand out for their versatility and susceptibility [15].

For this reason, this work aimed to evaluate the acute toxicity of the essential oils from *E. globulus*, *C. citratus*, and *M. piperita*, and DEET and cypermethrin against the isopod *Porcellio laevis.*

## 2. Material and Methods

### 2.1. Experimental Type and Design

A preclinical experimental study with a factorial (3Fa × 4Fb), controlled, randomized, and single‐blind (outcome assessment) arrangement was conducted. The experimental unit was the specimen of the isopod *P. laevis*. The sample, based on a previous study [15], consisted of 420 specimens (three essential oils × four concentrations × three replicates × 10 specimens) + (two controls × three replicates × 10 specimens) (Table 1).

**Table 1:** Experimental design.

Groups	Factor A	Factor B (%)	Outcome
Essential oils (*n* = 360)	∗ *Eucalyptus globulus* ∗ *Cymbopogon citratus* ∗ *Mentha piperita*	0.0	Mortality (%)
		0.1	
		1.0	
		10.0	
Cypermethrin (*n* = 60)		0.1	
DEET (*n* = 60)		10	

*Note:* DEET: N,N‐diethyl‐meta‐toluamide.

The chosen doses of essential oils were based on previous efficacy studies and a pilot test [2]. Furthermore, they include the concentrations of the synthetic comparator compounds. Regarding DEET, 10.0% is the most used concentration in commercial repellents in Latin America, and regarding cypermethrin, 0.1% is the recommended concentration for agricultural and public health use for vector control in Peru.

### 2.2. Biological Material

The three essential oils were extracted from E. *globulus, C. citratus*, *and M. piperita* leaves, selected according to their potential repellent use, protection time, and extraction yield based on a previous local study [2]. 50 mL of each oil was extracted by the steam stripping technique at the Organic Chemistry Laboratory of the School of Chemical Engineering and Food Industries of the Universidad Nacional Pedro Ruiz Gallo. The plant material was collected from two districts of the province of Chiclayo. Table 2 shows the geographical coordinates of the collection site and the botanical characteristics.

**Table 2:** Botanical and collection site characteristics of the biological material.

Scientific name (common name)	Family	Type of plant	Local use	Collection site (DMS)
*Eucalyptus globulus Labillardiere* (eucalyptus)	*Myrtaceae*	Tree	Ornamental, timber, and medicinal	Monsefú (−6.847791, −79.818263)
*Mentha piperita* L. (mint)	*Lamiaceae*	Herb	Medicinal and food	Reque (−6.839215, −79.786216)
*Cymbopogon citratus* (lemon verbena)	*Poaceae*	Herb	Food and medicinal	Reque (−6.839215, −79.786216)

*Note:* DMS = geographic coordinate system degrees, minutes, and seconds.

The specimens from which the colony of *P. laevis* (chanchito de tierra) was established were collected from agricultural areas in the district of Reque, Chiclayo (MSD: −6.839215, −79.786216). Several specimens from the colony were sent for identification and storage at the Klaus Raven Buller Entomology Museum of the Universidad Nacional Agraria La Molina (Batch 22–2024). The environmental and feeding conditions for the breeding and the maintenance of the isopods were established following the recommendations of Iannacone and Alvariño [15]. The colony was kept in 30 × 15 × 10 cm mesh‐covered plastic containers at the animal research facility (Biotherium) of the Hospital Regional Lambayeque. The substrate in each container was moistened daily using a water sprayer. Humidity conditions were also maintained at 70%–90%, with an ambient temperature of 25 ± 2° and a photoperiod of 12 h of light and 12 h of darkness.

Pregnant females, in groups of 10, were separated into exclusive containers to monitor births daily. The date of birth of each litter was recorded. Interdaily length measurements were then taken on the juveniles.

### 2.3. Evaluation of Acute Toxicity

Acute toxicity was evaluated using the percentage of mortality of *P. laevis* exposed to four concentrations of the essential oils diluted in extra virgin olive oil (0.0%, 0.1%, 1%, and 10%) and at three different times (3, 24, and 48 h). In addition, two positive controls were used: a farm insecticide (cypermethrin 0.1%) and a commercial synthetic repellent (DEET 10%). For the trial, 1 mL of each concentration was placed in 50‐mL glass beakers containing 10 g of sterilized farm soil. Subsequently, the dilutions were mixed with the substrate using a spatula until a homogeneous distribution was obtained. Finally, 10 young specimens, 2–3‐mm‐long, were added to the substrate, assigned by simple randomization to each experimental group. The tests were carried out in a laboratory with similar environmental conditions to the Biotherium, maintaining a minimum separation of 1 m between the beakers of each group. Mortality was evaluated at 3, 24, and 48 h. A specimen was considered dead when its pereopods were in a dorsal position and it did not move for 15 s of observation under the stereoscope [15].

### 2.4. Statistical Analysis

Data were recorded in a Microsoft Office Excel 2019 spreadsheet. Descriptive statistics were performed by calculating median and interquartile range for mortality in each experimental group and concentration tested. Inferential statistics were also performed to compare mortality between groups, concentration, and exposure time using Kruskal–Wallis nonparametric analysis of variance and Dunn’s test for multiple comparisons. The lethal concentration 50 (LC50) and 90 (LC90) of the essential oils in the experimental model were calculated by probit regression. A confidence level of 95% and statistical significance level of *p* < 0.050 were considered. The statistical software InfoStat/E was used for the analyses.

### 2.5. Ethical Considerations

The study was approved by the Institutional Ethics Committee for the Use of Animals (CEIPUA, for its Spanish initials) of the Hospital Regional Lambayeque, with certificate no. 1‐2024. The ethical principles of research and the standards for animal care were declared to be known and observed throughout the research process. The isopod specimens that participated in the trials were euthanized using 1% cypermethrin aerosol at the end of the trials. Study data are available upon request from the researchers.

## 3. Results

**Figure 1:**
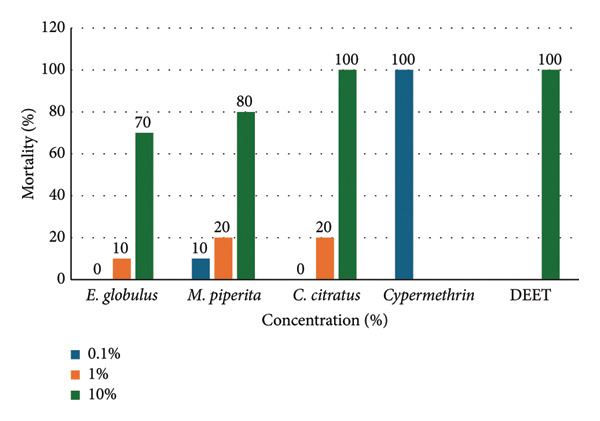
Comparative mortality of *Porcellio laevis* exposed to different concentrations of essential oils, cypermethrin at 0.1%, and DEET at 10%.

Table 3 and Figure 1 show the mortality of *P. laevis* exposed to different essential oils, cypermethrin (synthetic insecticide), and DEET (synthetic repellent). Essential oils showed lower toxicity at concentrations of 0.1% and 1.0% compared to cypermethrin and DEET, which are 100% lethal at the concentrations recommended for their effectiveness. However, essential oils at 10% concentration reached median mortality rates greater than 70%.

**Table 3:** Percentage of mortality of *Porcellio laevis* exposed to different concentrations of essential oils from *Eucalyptus globulus, Mentha piperita*, and *Cymbopogon citratus*, and cypermethrin and DEET.

Group	*N*	Concentration (%)		
		0.1	1.0	10.0
		Med (Q1–Q3)	Med (Q1–Q3)	Med (Q1–Q3)
*Eucalyptus globulus*	9	0 (0–10)	10 (0–10)	70 (20–70)
*Mentha piperita*	9	10 (0–20)	20 (10–30)	80 (30–100)
*Cymbopogon citratus*	9	0 (0–20)	20 (10–40)	100 (60–100)
Cypermethrin	9	100 (80–100)	nd	nd
DEET	9	nd	nd	100 (100–100)

*Note:* DEET: N,N‐diethyl‐meta‐toluamide; Med (Q1–Q3) = median (interquartile range).

Abbreviation: nd = no data.

In effect, the three essential oils showed similar toxicity to each other and equal to that in the negative control (10%–30% mortality), while they had significantly lower toxicity compared to cypermethrin and DEET (100% mortality). On the other hand, as for the concentrations tested, it was evidenced that 0.1% and 1.0% concentrations of the essential oils showed no toxicity since the mortality was similar to that in the negative control (10%). However, the 10.0% concentration of the essential oils caused a mortality of about 70%. Regarding the exposure times, it was observed that at 3 h, there was no toxic effect (0% mortality) on the animal model, but there was at 24 and 48 h. No difference was observed in these last two times (Table 4).

**Table 4:** Evaluation of the toxic effect (mortality) of the essential oils from *Eucalyptus globulus*, *Mentha piperita*, and *Cymbopogon citratus* on *Porcellio laevis* exposed to different concentrations and times.

Group	*n*	Median	Ranges	*p* value
Negative control	9	10	36.6 a	< 0.001
*Eucalyptus globulus*	27	10	40.8 a	
*Mentha piperita*	27	20	49.2 a	
*Cymbopogon citratus*	27	30	54.2 a	
Cypermethrin	9	100	89.6 b	
DEET	9	100	95.5 b	

*Concentration (%)*				
0.0	9	10	36.6 a	< 0.001
0.1	27	10	28.8 a	
1.0	27	10	40.1 a	
10.0	27	70	75.2 b	

*Exposure time (h)*				
3	36	0	35.7 a	< 0.001
24	36	20	57.9 b	
48	36	40	69.7 b	

*Note:* a, b ranges with unequal letters are significantly different (Dunn’s test, *p* < 0.05); DEET: N,N‐diethyl‐meta‐toluamide. Negative control = extra virgin olive oil; Kruskal–Wallis ANOVA *p* value.

Table 5 shows the LC50 and LC90 of the essential oils in the animal model, with concentrations higher than 12% being necessary to affect 90% of the specimens in 24 h.

**Table 5:** Lethal concentrations 50 (LC50) and 90 (LC90) of the essential oils from *Eucalyptus globulus, Mentha piperita*, and *Cymbopogon citratus* in *Porcellio laevis* at 24 h of exposure.

Group	LC50 (CI 95%)	LC90 (CI 95%)
*Eucalyptus globulus*	7.8 (5.2–9.8)	14.3 (12.3–16.8)
*Mentha piperita*	6.1 (4.9–7.4)	12.6 (10.8–15.0)
*Cymbopogon citratus*	9.8 (8.9–10.6)	12.2 (11.2–16.0)

## 4. Discussion

These results suggested the potential use of *E. globulus, M. piperita*, and *C. citratus* essential oils in public health due to their lower toxicity compared to commercial synthetic compounds such as 10% DEET (repellent) and 0.1% cypermethrin (insecticide for agricultural, public health, and industrial use).

The lower acute toxicity of essential oils in experimental animal models can be attributed to the natural origin of their different aromatic and aliphatic compounds [16]. This toxicity could be explained by their hydrophobicity, which allows them to adhere to membranes, affecting their pressure and permeability. In addition, several mechanisms involve their olfactory receptors [3]. The inhibitory action of γ‐aminobutyric acid receptors or interference with acetylcholinesterase activity has also been described; both are key neurotransmitters of the insect’s central nervous system [8]. However, the exact pathophysiological mechanisms of their effect on invertebrates are unknown.

Furthermore, the high toxicity of 10% DEET observed could be due to its action on octopaminergic synapses to induce neuroexcitation [17]. The high toxicity of cypermethrin in the isopod is explained by the interference with sodium channels in neurons, overexciting and finally paralyzing the invertebrate. Therefore, this pyrethroid is neurotoxic, and its lethality is dose‐dependent [18]. Also, adverse effects on the immune and nervous systems have been reported in humans through exposure by inhalation, ingestion, or skin contact [19].

The worldwide interest in the prevention of mosquito‐borne diseases has led to the increasing use of repellents or insecticides, mostly synthetic [3]. In this context, essential oils have become an environmentally sustainable alternative in the pharmaceutical, cosmetic, and food industries [8]. However, it is important to guarantee that they are safe, which is carried out through different experimental models, mainly in isopods of the genus *Porcellio*, that have also been used to evaluate the toxicity of pesticides and environmental pollutants due to their versatility and susceptibility [14, 20]. Furthermore, it is recommended to replicate these toxicity assays in mammalian cell lines and other animal models to describe the lethal and sublethal effects of essential oils.

All the essential oils tested in this study needed more than a 6% (60,000 mg/L) concentration to achieve a 50% mortality rate of the isopods. Although there are no previous studies in this experimental model, other studies conducted on *A. aegypti* larvae reported that an insecticidal effect was observed at 5 mg/L and above [21], suggesting greater toxic activity in this type of insect at its larval stage. Other evaluations of cypermethrin have reported LC50s of 0.01103 mg/L and 0.00207 mg/L for adult *A. aegypti*, Orotina strain and Rockefeller strain, respectively [22]. Likewise, an LC50 of DEET has been reported at 2.3% also in adult *A. aegypti* [17]. These data show the lower toxicity of the essential oils compared to the synthetic compounds mentioned above.

Finally, as with currently used commercial synthetic repellents, the frequency of application of essential oil–based repellents will depend on the formulated concentration, the repellent’s presentation (lotion, gel, or cream), and the observed protection time [23]. In this regard, a previous study reported protection times of 120–180 min for the essential oils of *E. globulus*, *M. piperita*, and *C. citratus* at concentrations greater than 25% [2]. Additionally, 25% DEET has been reported to have a longer protection time than 25% eucalyptus oil [23].

Another aspect to consider regarding essential oil–based repellents is their cost–benefit ratio and consumer acceptability compared to synthetic repellents. Furthermore, their high volatility could be mitigated through encapsulation and emulsification techniques [24]. This will require specific market studies that consider the different origins of the botanical material and the concentrations used in the formulations. Likewise, the environmental impact and the increasing dosage of synthetic repellents, which has reached 30% in the case of DEET, must be considered [23].

This study had the limitation that phytochemical characterization of the essential oils was not performed for logistical reasons. It was also not possible to describe the pathophysiology of damage in the isopod experimental model. Further toxicity studies in other experimental models, such as mammalian cell lines, will be needed to strengthen the evidence and extrapolate to human safety. However, it is one of the first studies to evaluate the safety of these types of plant compounds with potential pharmaceutical use and impact on public health.

It is concluded that the essential oils from *E. globulus*, *C. citratus*, and *M. piperita* showed similar acute toxicity to each other but significantly lower compared to DEET and cypermethrin, tested in the invertebrate model of the isopod *P. laevis*. It is suggested to complement with studies on the pathophysiological mechanisms of the toxicity of essential oils in invertebrate models, as well as chronic toxicity in vertebrate models.

## Disclosure

All authors approved this final version of the report and are responsible for the results reported.

## Conflicts of Interest

The authors declare no conflicts of interest.

## Author Contributions

All authors contributed equally to the study design and sample and data collection for the study. Heber Silva‐Díaz performed the data analysis and interpretation. All authors drafted the manuscript and performed the critical review.

## Funding

This study was supported by the Universidad de San Martín de Porres, Northern campus. The project was approved with code number E2110202023009. Hospital Regional Lambayeque contributed nonmonetary support.

## Data Availability

The data that support the findings of this study are available from the corresponding author upon reasonable request.
